# Factors associated with high risky sexual behavior among students engaging in casual heterosexual activity: a cross-sectional study in Zhejiang Province, Eastern China

**DOI:** 10.3389/fpubh.2025.1643337

**Published:** 2025-11-24

**Authors:** Zhongrong Yang, Qiaoqin Ma, Weiyong Chen, Wanjun Chen, Xin Zhou

**Affiliations:** 1 Department of HIV/TB Control and Prevention, Huzhou Center for Disease Control and Prevention, Huzhou, Zhejiang, China; 2 Department of HIV/STD Control and Prevention, Zhejiang Provincial Center for Disease Control and Prevention, Hangzhou, Zhejiang, China; 3 Zhejiang Key Lab of Vaccine, Infectious Disease Prevention and Control, Hangzhou, Zhejiang, China

**Keywords:** AIDS, HIV, risk factors, casual sex, college students, HIV prevention

## Abstract

**Objective:**

This study aimed to investigate the factors associated with engagement in high risky sexual behavior among college students who have engaged in casual heterosexual behaviors.

**Method:**

A cross-sectional study was performed in Zhejiang Province, Eastern China between November and December 2020. Stratified cluster sampling method was used for the survey, and demographic and behavioral data was collected through questionnaires for statistical analysis.

**Results:**

In total, 2,581 university students self-reported engaging in heterosexual behavior in the previous year, accounting for 6.15% (2,581/41,940) of the student population. From this group, 425 college students who reported engaging in casual heterosexual behaviors in the past year were included, of whom 53 (12.47%, 53/425) engaged in high risky sexual behavior. The results of the multivariable logistic regression analysis indicated that the participants who accepted sexual behavior with men who had sex with men (MSM), those who involved in commercial sexual activities with monetary transactions in the past year were more likely to have engaged in high risky sexual behavior. Additionally, the participants who consistently used condoms during sexual activity with casual sexual partners and those who used condoms occasionally were less likely to have engaged in high risky sexual behavior.

**Conclusion:**

This study identified several key factors associated with high risky sexual behavior among college students engaging in casual heterosexual experience. It is advisable to incorporate sexual risk awareness and sex moral education into health programs for this population. We also need enhance students’ understanding of HIV transmission risks and promote consistent condom use, in order to reduce the likelihood of HIV infection.

## Introduction

HIV/AIDS causes persistent damage to the immune systems of patients, leading to the development of various diseases and severe complications that significantly affect public health (1, 2). In recent years, the increase in HIV/AIDS infections among young students has garnered considerable attention, particularly in China, where the epidemic has been characterized by a rapid rise in infection rates (3). Young students have emerged as a key focus group for the prevention and control of HIV/AIDS. Approximately 3,000 cases of university students contracting HIV are reported in China annually, with sexual activity being the primary route of transmission (4). University students are in a period of sexual activity and often engage in active sexual activity influenced by societal openness and attitudes towards sex, resulting in common occurrences of premarital sexual activity. However, a significant proportion of these young individuals have inadequate knowledge of sexual health and HIV transmission routes, highlighting the necessity for comprehensive and accurate sex education programs to address risky behaviors, such as engaging in unprotected sex or having multiple sexual partners, which can ultimately increase the possibility of HIV infection (5, 6).

In recent years, casual heterosexual behavior among university students (such as non fixed partner sexual behavior) has gradually become a focus of sexual health research, and anal sex behavior is particularly important due to its high-risky characteristics (7, 8). About 4% of college students reported having anal sex in the past 3 months, with a higher proportion in non fixed partner relationships (9). Furthermore, anal intercourse poses a higher risk for HIV/sexually transmitted disease (STD) transmission than vaginal and oral sex, with varying proportions of anal sex occurrences observed across different sexes (10, 11). Engaging in anal sex carries potential health risks because of the fragile nature of anal tissues; improper techniques and force during anal intercourse can result in tissue damage and lacerations, making the anal region susceptible to sexually transmitted infections (such as HIV, gonorrhea, and syphilis) (12, 13). Additionally, involvement in anal sex may lead to anxiety (14). Therefore, gaining insight into the patterns of anal sexual behavior among university students engaging in transient heterosexual activities is pivotal to combat the spread of HIV within this demographic.

Although studies have revealed the epidemiological characteristics of HIV/STD related risky behaviors among college students (1, 15, 16), there is still a significant gap in exploring the influencing factors of specific sexual behavior patterns, such as anal sex behavior among heterosexual students. Many existing studies are limited to describing the incidence of behavior (10, 11), and lack in-depth analysis of multidimensional influencing mechanisms. This study aimed to investigate the characteristics and factors associated with anal sexual behavior among college students engaged in casual heterosexual activities. We collected relevant data for analysis through a cross-sectional study of university students in Zhejiang Province, Eastern China. This study contributes to a better understanding of the characteristics of anal sexual behavior among college students and provides a scientific basis for the development of targeted prevention and intervention measures.

## Materials and methods

### Study design

This study used a cross-sectional survey to collect data. A survey was conducted among university students from 15 institutions in 11 prefecture-level cities in Zhejiang Province between November and December 2020. The universities were selected based on recommendations from the local center for disease control and prevention, with five universities from Hangzhou and one from each of the remaining 10 cities. A stratified cluster sampling technique was employed for the survey process, beginning with the selection of three departments (each with a minimum of 800 students) from each university using a random number generator. Subsequently, classes were randomly chosen from each academic year, ensuring a minimum of 200 students per grade for the four-year programs and 267 students per grade for the three-year programs. All the students in the selected classes participated in the survey. On-campus students completed an electronic survey questionnaire provided by their instructors, whereas off-campus students received a survey link and were instructed to complete the questionnaire independently as directed on the first page.

### Participants

The sample size calculation for this study was based on a cross-sectional survey sampling method. The prevalence of sexual behavior among college students was estimated to be around 15% (*p* = 0.15). By applying the formula *n* = 400*Q/P, where Q = 1−P, the necessary sample size for the study was determined to be 2,267 participants. In total, 42,380 students participated in the survey, the response rate of those students invited to participate in this study is 100%. The inclusion criteria mainly include college students who have only engaged in heterosexual activity in the past year, as well as those who have given informed consent to participate in the study. The exclusion criteria mainly exclude college students who have engaged in both heterosexual and homosexual activity in the past year.

**Figure 1:**
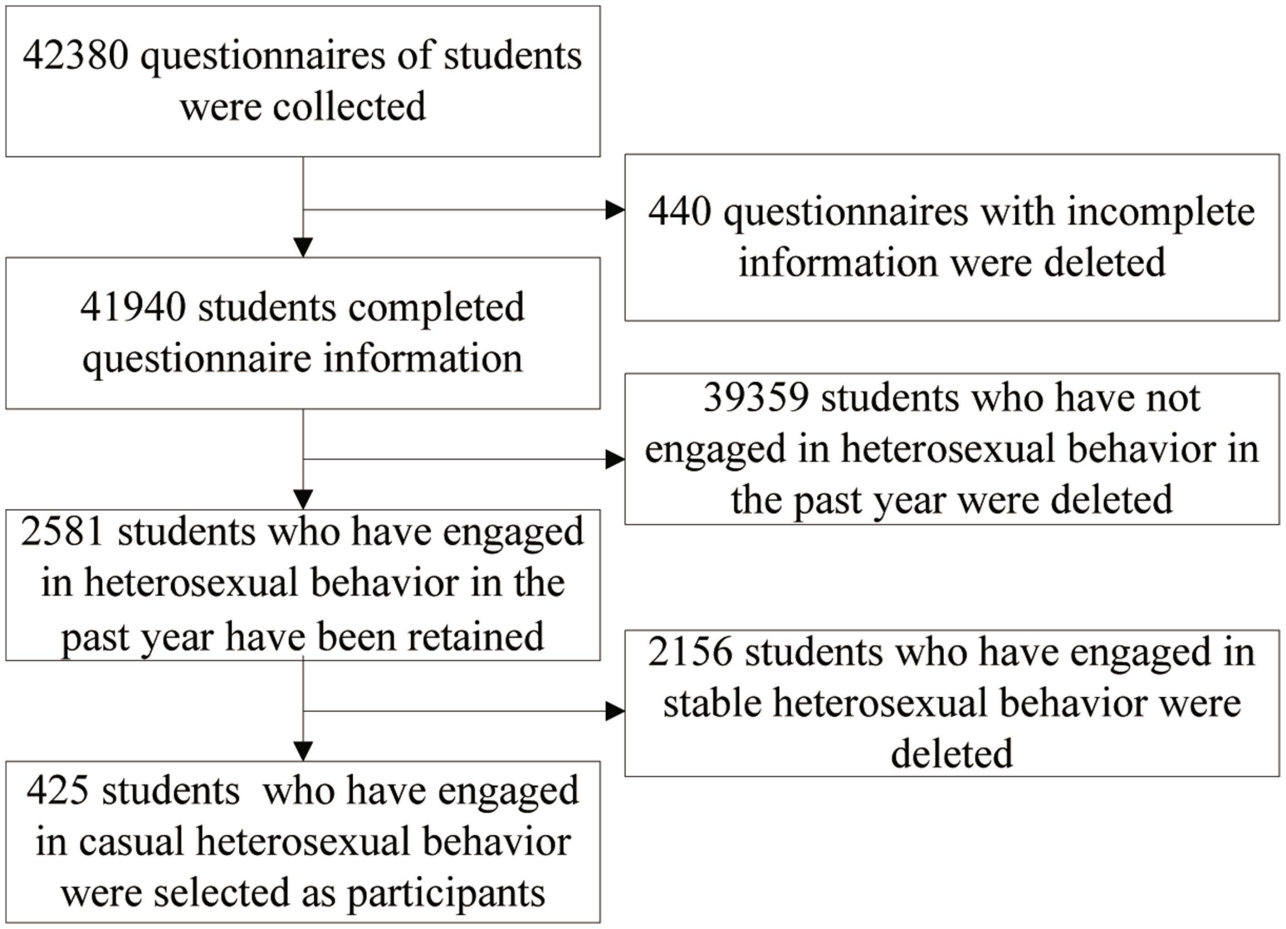
The flowchart for the inclusion and exclusion process.

After eliminating 440 incomplete questionnaires, 41,940 responses were analyzed. Among them, 2,581 university students self-reported engaging in heterosexual behavior in the previous year, accounting for 6.15% (2,581/41,940) of the student population. From this group, 425 university students who reported casual heterosexual behavior in the past year were selected as participants, representing 16.47% (425/2,581) of university students engaging in such behaviors (Figure 1).

### Ethics and consent

The study protocol was approved by the ethics committee of the Zhejiang Provincial Center for Disease Control and Prevention, complied with the Declaration of Helsinki, and was performed in accordance with the relevant guidelines and regulations of the ethics committee of the Zhejiang Provincial Center for Disease Control and Prevention (No. 2018-036). All the participants provided written informed consent.

### Contents of the questionnaire

The survey questionnaire was developed based on a review of the related literature (17, 18), discussions within the research team. The survey questionnaire included general demographic characteristics, attitudes towards sexuality, sexual behavioral characteristics, HIV testing status, HIV risk perception, online dating, confidence in condom use, and other related topics (19, 20). The survey was conducted anonymously using a standardized questionnaire, and the surveyors comprised professional staff from the local disease control and prevention centers and counselors from the surveyed university classes. They received uniform training before the survey. Prior to the survey, the surveyors explained the purpose, significance, methods, and privacy protection policy to the students. This information was included in the introduction of the survey questionnaire. Participants were informed that the survey was anonymous, aimed at formulating HIV and STDs prevention strategies for students, and that only group data would be analyzed.

### Definition of research groups and related indicators

The dependent variables is refer to participants who reported participating in casual heterosexual activities were divided into two groups based on whether they had engaged in high risky sexual behavior (such as anal sex) in the past year: the anal intercourse group and the non-anal intercourse group. The independent variables included demographic characteristics, knowledge of HIV/AIDS prevention, attitude towards sex, perception of HIV risk, effectiveness of public health campaigns, and sexual behavioral traits. High risky sexual behavior is refer to anal sex.

### Quality control

The survey team comprised professionals from the local disease prevention and control center and counselors from surveyed classes in different universities. They underwent standardized training before conducting anonymous surveys using a unified questionnaire. Before the survey, the investigators elucidated the purpose, significance, survey methods, and privacy policies of the study to the participants, with relevant details outlined in the initial segment of the questionnaire. Participants were informed that the survey aimed to formulate strategies for the prevention of AIDS and STDs among college students. The survey was anonymized, and the analysis focused solely on aggregate data rather than on individual data.

### Statistical analysis

SPSS software (version 23.0; SPSS Inc., Chicago, IL, United States) was used for data analysis. Variables such as age, grade, and sexual behavior characteristics are expressed as means, composition ratios, or rates. This study examined the occurrence of high risky sexual behavior (such as anal sex behaviors) in the past year based on self-reported data, with the dependent variable being whether such activities occurred. Logistic regression analysis was performed to investigate the factors associated with anal sex behaviors among the participants. The model for multivariate logistic regression included age, sex, household registration, and variables with a significance level of *p* < 0.20 from the univariate analysis. Statistical significance was set at *p* < 0.05. We performed a goodness of fit test for the multivariate logistic regression model, evaluating its explanatory capacity and predictive performance against observed data, and the coefficient of determination (R-squared value) indicates the proportion of variance explained by the model.

## Results

### General demographic characteristics

A total of 425 college students who engaged in casual heterosexual sex in the past year were included, among whom 53 (12.5%) reported engaging in high risky sexual behavior such as anal sexual behaviors, with an average age of 20.19 ± 1.36 years. The remaining 372 students (87.5%) did not report anal sexual behavior, and had an average age of 20.01 ± 1.22 years. In the group of students who engaged in anal sexual behavior, 32.1% were ≤19 years old, 88.7% were men, 43.4% were from other provinces, and 62.3% attended regular higher education institutions. Differences in variables such as age, sex, household registration, and school type between the two groups of students were not statistically significant (Table 1).

**Table 1:** Demographic characteristics of participants.

Variables	Anal intercourse group (*n* = 53)		Non-anal intercourse group (*n* = 372)		χ^2^	*P*
	*n*	%	*n*	%		
Age (yrs.)						
Less than or equal to 19	17	32.1	131	35.2	2.943	0.230
20–21	26	49.1	201	54.0		
Greater than or equal to 22	10	18.9	40	10.8		
Sex					2.472	0.116
Female	6	11.3	76	20.4		
Male	47	88.7	296	79.6		
Province of household registration					1.961	0.161
Zhejiang Province	30	56.6	247	66.4		
Non-Zhejiang Province	23	43.4	125	33.6		
The type of school					0.191	0.662
Higher vocational college	20	37.3	129	34.7		
Ordinary higher education institutions	33	62.3	243	65.3		

### Association factors analysis of high risky sexual behavior among participants

In the univariate analysis (Table 2), participants who had accepted sexual behavior with men who have sex with men (MSM) (crude odds ratio [COR] = 3.35), those who engaged in commercial sexual activities with monetary transactions in the past year (COR = 7.94), and those feeling at risk of contracting HIV (COR = 2.51) suggested a higher possibility of engaging in anal sex. Understanding that “consistently using condoms correctly can reduce the risk of HIV infection and transmission” (COR = 0.25), “HIV counseling and testing should be actively sought after high-risk sexual behaviors” (COR = 0.26), using condoms consistently (COR = 0.05), or using condoms occasionally (COR = 0.15) with casual partners suggested a lower possibility of engaging in high risky sexual behavior.

**Table 2:** Association factors analysis with high risky sexual behavior among 425 participants.

Variables	Anal intercourse group (*n* = 53)		Non-anal intercourse group (*n* = 372)		Univariable analysis		Multivariable analysis	
	*n*	%	*n*	%	OR (95%CI)	*P*	OR (95%CI)	*P*
Age (yrs.)								
Less than or equal to 19	17	11.5	131	88.5	1		1	
20–21	26	11.5	201	88.5	1.00 (0.52–1.91)	0.992	0.70 (0.32–1.55)	0.382
Greater than or equal to 22	10	20.0	40	80.0	1.93 (0.82–4.54)	0.134	1.82 (0.61–5.39)	0.283
Sex								
Female	6	7.3	76	92.7	1		1	
Male	47	13.7	296	86.3	2.01 (0.83–4.88)	0.122	2.13 (0.70–6.51)	0.184
Province of household registration								
Zhejiang Province	30	10.8	247	89.2	1		1	
Non-Zhejiang Province	23	15.5	125	84.5	1.52 (0.85–2.72)	0.163	1.24 (0.59–2.60)	0.576
Whether consistently using condoms correctly can reduce the risk of HIV infection and transmission								
No/Do not know	9	33.3	18	66.7	1		1	
Yes	44	11.1	354	88.9	0.25 (0.11–0.59)	0.001	0.75 (0.12–4.51)	0.750
Whether HIV counseling and testing should be actively sought after high-risk sexual behaviors								
No/Do not know	7	33.3	14	66.7	1		1	
Yes	46	11.4	358	88.6	0.26 (0.10–0.67)	0.005	1.10 (0.14–8.77)	0.928
Whether learned about AIDS through the school network in the last year								
No	13	16.9	64	83.1	1		1	
Yes	40	11.5	308	88.5	0.64 (0.32–1.26)	0.198	0.54 (0.22–1.36)	0.192
Whether did the school provide HIV testing services								
No/Do not know	27	10.3	236	89.7	1		1	
Yes	26	16.0	136	84.0	1.67 (0.94–2.98)	0.082	1.87 (0.89–3.94)	0.101
Whether accepted sexual behavior with MSM^*^								
No/Do not know	27	8.5	289	91.5	1		1	
Yes	26	23.9	83	76.1	3.35 (1.86–6.06)	0.000	3.10 (1.37–7.04)	0.007
Condom use with casual sexual partners								
Never used	28	47.5	31	52.5	1		1	
Used occasionally	16	11.9	118	88.1	0.15 (0.07–0.31)	0.000	0.24 (0.10–0.58)	0.002
Used consistently	9	3.9	223	96.1	0.05 (0.02–0.10)	0.000	0.08 (0.03–0.20)	<0.001
Whether casual sexual partners get to know each other through the Internet								
No	32	10.8	263	89.2	1		1	
Yes	21	16.2	109	83.8	1.58 (0.87–2.87)	0.129	0.66 (0.31–1.43)	0.295
Whether engaged in commercial sexual behaviors involving monetary transactions in the past year								
No	25	7.1	326	92.9	1		1	
Yes	28	37.8	46	62.2	7.94 (4.26–14.78)	0.000	3.09 (1.34–7.10)	0.008
Whether thought that you are at risk of contracting HIV								
No/Do not know	44	11.3	344	88.7	1		1	
Yes	9	24.3	28	75.7	2.51 (1.11–5.67)	0.026	1.03 (0.34–3.12)	0.952
Whether received voluntary counseling and testing in the past year								
No	44	11.5	339	88.5	1		1	
Yes	9	21.4	33	78.6	2.10 (0.94–4.68)	0.069	0.96 (0.35–2.69)	0.946

*MSM, men who have sex with men.

The results of multivariate logistic analysis (Table 2) revealed that the possibility of engaging in high risky sexual behavior was 210% higher for participants who had accepted sexual behavior with MSM (Adjusted Odds Ratio [AOR]: 3.10; 95% confidence interval [CI]: 1.37–7.04) and 209% higher for those involved in commercial sexual activities with monetary transactions in the past year (AOR: 3.09; 95% CI: 1.34–7.10). Additionally, the possibility of high risky sexual behavior decreased by 76% for participants who consistently used condoms with casual sexual partners (AOR: 0.24; 95% CI: 0.10–0.58) and by 92% for those who used condoms occasionally (AOR: 0.08; 95% CI: 0.03–0.20).

## Discussion

Recently, the prevalence of AIDS among college students has concerningly increased. Therefore, enhancing health education efforts, promoting safe sexual practices, and advocating widespread AIDS testing is crucial to prevent infection (21, 22). This cross-sectional survey on HIV/AIDS prevention was conducted among university students across Zhejiang Province, providing a representative assessment of sexual behavior prevalence and associated determinants among the provincial collegiate population. Implemented in late 2020, the study revealed a self-reported sexual activity rate of 6.15% (2,581/41,940) among surveyed students. This relatively low prevalence may be partially attributable to COVID-19 containment policies, particularly quarantine measures and mobility restrictions during pandemic control periods, which likely reduced interpersonal interactions and contributed to decreased sexual encounters within the population. The findings revealed that of 425 students involved in such relationships within the past year, 12.5% reported engaging in anal intercourse, highlighting the potential risk of the transmission of STDs, including HIV.

The risk of HIV infection for the recipient during anal intercourse is significantly higher than that during vaginal intercourse, varying from several times to >10 times, because of physiological factors (23). The findings of this study indicate that, among college students who engaged in casual heterosexual activities in the past year, the prevalence of high risky sexual behavior was 13.7% among male students and 7.3% among female students. Various factors, including sex perceptions, level of sexual education, sexual preferences, and cultural backgrounds may influence the occurrence of this sexual behavior. It is essential to highlight that engaging in high risky sexual behavior can heighten the risk of contracting STDs such as HIV. Therefore, emphasizing sex education and protective measures as vital strategies for mitigating the spread of such diseases is important. Additionally, continual monitoring of behaviors such as condom usage and sexual activities is crucial.

Anal intercourse has been associated with an elevated risk of contracting sexually transmitted infections, particularly in cases where protective measures are inadequately implemented. The findings of this study indicated that the prevalence of high risky sexual behavior is 3.10 times higher among participants whom accepted sexual behavior with MSM. These results underscore the necessity for enhanced sexual education interventions aimed at disseminating knowledge on same-sex sexual practices and fostering a culture of sexual safety awareness. Such interventions should encompass proper condom use during all sexual encounters to equip students with the requisite understanding of the associated health risks and preventive strategies. Consequently, forthcoming AIDS prevention initiatives must underscore the communication of sexual risks, improve the comprehension of disease vulnerabilities and HIV screening rates in students, and advocate for responsible partner selection to mitigate the risk of HIV transmission.

The occurrence of high risky sexual behavior among heterosexual groups is influenced by various factors, including curiosity, pursuit of sexual pleasure, and contraception (24). Individuals engaging in high risky sexual behavior are more likely to seek partners who are HIV-positive or involved in sex work (25). This study revealed a 209% increase in high risky sexual behavior among college students involved in commercial sex work in the past year. Commercial sex work poses a heightened risk of HIV or STDs, as money often serves as a means of obtaining sexual gratification, potentially leading individuals to explore new sexual practices, such as anal sex. These findings underscore the need to address the impact of commercial sex work on the sexual health of college students. Efforts should be made to enhance sexual health education, promote safe sexual practices, and implement intervention strategies that target commercial sexual work within college communities. Consequently, HIV/AIDS health education in academic settings should prioritize instilling proper sexual ethics and concepts to mitigate the occurrence of unsafe commercial sexual activities.

The consistent use of condoms during sexual activity serves as a crucial measure for sexual protection, effectively combating the spread of HIV (26). The data analysis revealed a direct correlation between high risky sexual behavior and lower condom usage rates, highlighting the need for increased awareness and preventive measures in such scenarios (27). Given the potential for mucosal damage and heightened vulnerability to STDs associated with high risky sexual behavior, the use of condoms has emerged as a pivotal strategy for mitigating these risks. Condoms act as a barrier, impeding direct contact with bodily fluids and pathogens, thereby significantly reducing the possibility of transmission of pathogens and safeguarding the health of all involved parties. The results of this study underscore the positive impact of condom use among university students during casual sexual engagement, notably decreasing the occurrence of high risky sexual behavior. Notably, the frequency of condom use among university students engaging in casual sex remains suboptimal, with usage rates standing at a mere 54.6%. Particularly concerning is the disparity in condom use between the group that engaged in high risky sexual behavior (17.0%) and the group that abstained from it (59.9%). Apart from lowering the risk of dissemination of STDs, condom use fosters a heightened sense of security and mutual respect in sexual interactions. By proactively choosing to use condoms, individuals cultivate a responsible attitude towards their sexual behavior, underscoring the importance of care, protection, and disease prevention while upholding the dignity of such interactions. Despite the significance of condoms in sexual health, numerous challenges persist regarding their widespread adoption. Instances of reluctance of condom use among certain university students, stemming from inadequate sexual education or conservative ideologies, have contributed to diminished usage rates. Therefore, it is imperative that health education initiatives targeting university students should underscore individual accountability for personal health, intensify sex education efforts, and enhance the comprehension and acceptability of condoms among this demographic to bolster their utilization. The phenomenon of high risky sexual behavior among college students, particularly those engaged in casual heterosexual behaviors who may be at higher risk for unsafe practices, warrants significant attention. Higher education professionals must prioritize the prevention and management of related sexual risks in this population. Beyond disseminating HIV prevention knowledge and sexual health education through standard curricula, targeted interventions for subgroups with specific risk factors (as identified in this study) are crucial. Special attention should also be paid to the physiological and psychological health needs of these students (28).

## Study limitations

This study has several limitations. First, considering the cross-sectional nature of the survey, causal conclusions could not be drawn regarding the identified factors. We attempted to minimize the influence of potential confounding variables in the study design and performed thorough statistical analyses of the survey data using single and multiple logistic regressions to establish a more precise understanding of the relationships between factors, control for potential variables in the hypothesis, and draw careful inferences. Additionally, the reliance on self-reported data from participants, particularly on sensitive topics such as sexual behavior, introduces the possibility of recall bias or social desirability bias. Moreover, the presence of social desirability bias could further affect the reported behaviors and attitudes towards sexual practices, including anal intercourse, potentially influencing the study outcomes. We have also conducted a goodness of fit test on the multivariate logistic regression model, and the R-squared value of 0.502 suggested a less strong fit. This study used a self-administered questionnaire to collect sexual behavior data, where “anal sex behavior” is defined as the anal sexual contact that the respondents actively or passively participated in within the past 12 months. However, due to limitations in questionnaire design, there was no further differentiation of the contexts in which anal sex occurs (such as heterosexual/homosexual partners, active/passive roles). This study is a cross-sectional investigation conducted exclusively within Zhejiang Province. However, generalizing the findings to the entire nation would require multi-center prospective cohort follow-up studies to further investigate the relevant influencing factors. The limitation of this study is that it did not conduct a multidimensional analysis of the interactions and potential mechanisms among factors related to anal sex behavior, such as social cognition, partner dynamics, or cultural norms influencing pathways. It is recommended to combine structural equation modeling or mediation analysis to quantify the correlation between variables in the future, and supplement quantitative data through qualitative interviews. Using social cognitive theory or health behavior models as frameworks, systematically reveal how various factors affect behavioral decisions through mediating variables such as risk perception and self-efficacy, and thus construct a more complete explanatory model. The exploration of mechanisms driven by this theory can help enhance the scientificity of intervention strategies.

## Conclusion

This study found that a certain proportion of college students in the Zhejiang Province who engaged in casual heterosexual behaviors also engaged in high risky sexual behavior. Factors associated with engaging in high risky sexual behavior among this group included acceptance of sexual behavior with MSM, involvement in commercial sex with monetary transactions, and condom use with casual sexual partners. Therefore, increasing sexual risk awareness and sex moral education in health education programs for this population is advisable. This approach aims to enhance the awareness of HIV infection risks among college students, promote the use of condoms, and consequently reduce the possibility of HIV transmission.

## Data Availability

The raw data supporting the conclusions of this article will be made available by the authors, without undue reservation.
